# Development and Validation of a Five-Gene Signature to Predict Relapse-Free Survival in Multiple Sclerosis

**DOI:** 10.3389/fneur.2020.579683

**Published:** 2020-12-03

**Authors:** Fei Ye, Jie Liang, Jiaoxing Li, Haiyan Li, Wenli Sheng

**Affiliations:** ^1^Department of Neurology, The First Affiliated Hospital, Sun Yat-sen University, Guangzhou, China; ^2^Guangdong Provincial Key Laboratory of Diagnosis and Treatment of Major Neurological Diseases, The First Affiliated Hospital, Sun Yat-sen University, Guangzhou, China; ^3^Department of Neurology, The Third Affiliated Hospital, Sun Yat-sen University, Guangzhou, China

**Keywords:** multiple sclerosis, relapse-free survival (RFS), gene signature, bioinformatic analysis, translational medicine

## Abstract

**Background:** Multiple sclerosis (MS) is an inflammatory and demyelinating disease of the central nervous system with a variable natural history of relapse and remission. Previous studies have found many differentially expressed genes (DEGs) in the peripheral blood of MS patients and healthy controls, but the value of these genes for predicting the risk of relapse remains elusive. Here we develop and validate an effective and noninvasive gene signature for predicting relapse-free survival (RFS) in MS patients.

**Methods:** Gene expression matrices were downloaded from Gene Expression Omnibus and ArrayExpress. DEGs in MS patients and healthy controls were screened in an integrated analysis of seven data sets. Candidate genes from a combination of protein–protein interaction and weighted correlation network analysis were used to identify key genes related to RFS. An independent data set (GSE15245) was randomized into training and test groups. Univariate and least absolute shrinkage and selection operator–Cox regression analyses were used in the training group to develop a gene signature. A nomogram incorporating independent risk factors was developed via multivariate Cox regression analyses. Kaplan–Meier methods, receiver-operating characteristic (ROC) curves, and Harrell's concordance index (C-index) were used to estimate the performance of the gene signature and nomogram. The test group was used for external validation.

**Results:** A five-gene signature comprising FTH1, GBP2, MYL6, NCOA4, and SRP9 was used to calculate risk scores to predict individual RFS. The risk score was an independent risk factor, and a nomogram incorporating clinical parameters was established. ROC curves and C-indices demonstrated great performance of these predictive tools in both the training and test groups.

**Conclusions:** The five-gene signature may be a reliable tool for assisting physicians in predicting RFS in clinical practice. We anticipate that these findings could not only facilitate personalized treatment for MS patients but also provide insight into the complex molecular mechanism of this disease.

## Introduction

Multiple sclerosis (MS) is a common chronic inflammatory and demyelinating disease of the central nervous system characterized by a highly variable natural course of relapse and remission (1). This hardly curable disease affects more than 2 million young adults around the world (1). Initial symptoms of dysfunction in the optic nerves, brainstem, or spinal cord may be diagnosed as clinically isolated syndrome, which develops into MS in ~85% of cases (2). Relapse, an essential feature of autoimmune diseases, including MS, is defined as a new onset or exacerbation of neurological dysfunction; it is usually followed by a period of remission with no disease activity. The underlying cause of relapse is not fully understood, but it is associated with a loss of axons and gray matter pathology (3). Current evidence suggests that the relapse rate is higher in younger patients and women. In addition, a shorter disease duration, a lower baseline Expanded Disability Status Scale score (4), radiological lesions, and pregnancy are risk factors for relapse and a poor outcome (5, 6). However, controversy surrounds the use of disease-modifying therapies to protect against relapses and improve progressive neurologic deterioration because of their long-term pharmacological effects and individual variants (1, 7, 8).

Many scientific studies have demonstrated differences in gene expression in peripheral whole blood or peripheral blood mononuclear cells (PBMCs) between MS patients and healthy subjects, but the value of these genes for predicting the prognosis of MS patients is unknown (9–14). Consequently, it is necessary to develop novel markers for predicting the occurrence of relapse and estimating the interval between relapses. Such a measurement could provide important information for physicians deciding on personalized therapeutic strategies for MS patients. The gene signature is a convenient predictive instrument based on differentially expressed genes (DEGs) that can be used to calculate a risk score to evaluate individual outcomes (15, 16). The purpose of our study was to develop and validate an effective and noninvasive prognostic gene signature for predicting the probability of relapse and remission period in MS patients via an integrated analysis of blood microarrays.

## Materials and Methods

### Data Downloading and Processing

**Figure 1:**
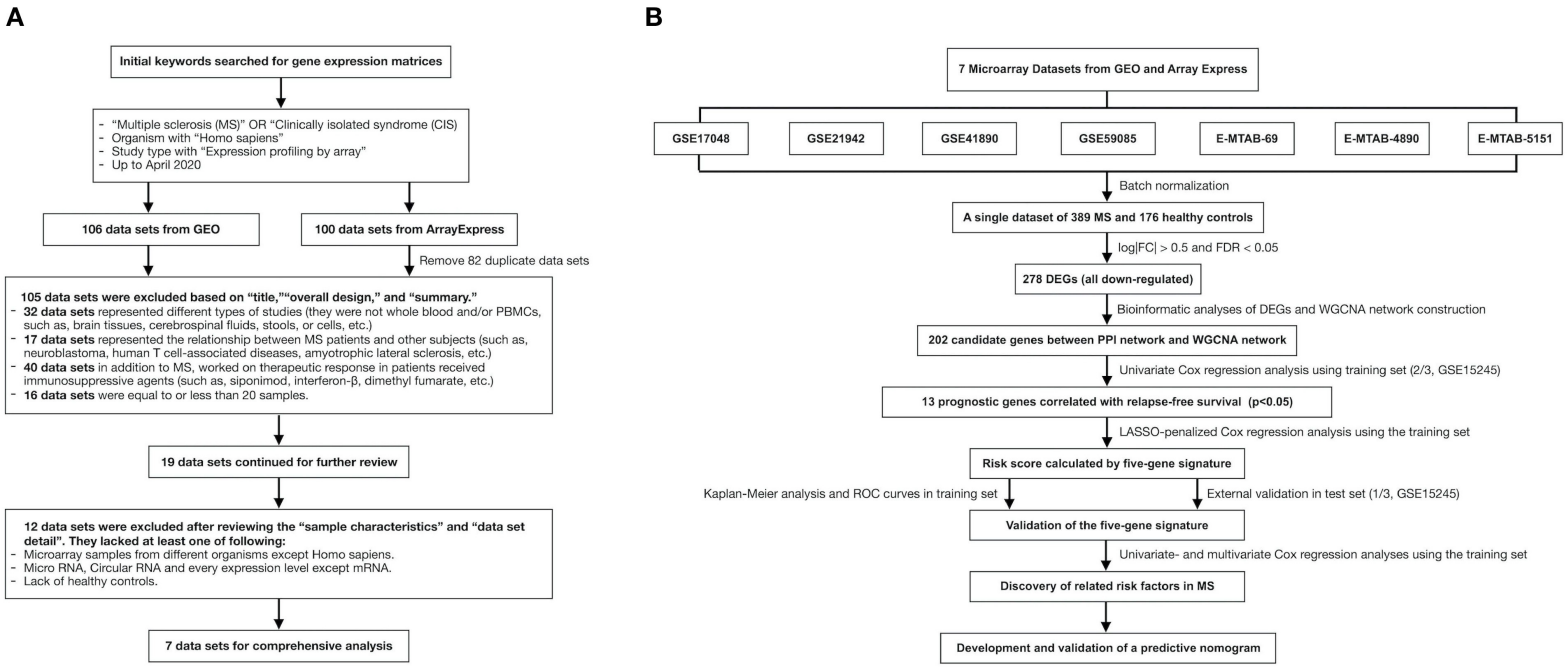
The flow charts of this study in detail. **(A)** A flow chart showing the process used to select the gene expression matrices. **(B)** A flow chart showing the process used to develop the predictive gene signature and nomogram of MS. GEO, Gene Expression Omnibus; MS, multiple sclerosis; FC, fold change; FDR, false discovery rate; DEG, differentially expressed gene; WGCNA, weighted correlation network analysis; PPI, protein–protein interaction; LASSO, least absolute shrinkage and selection operator; ROC, receiver-operating characteristic.

The data sets were searched and downloaded according to Figure 1A. We searched for gene expression matrices and clinical information using the keywords “multiple sclerosis,” “clinically isolated syndrome,” “MS,” and “CIS” in Gene Expression Omnibus (https://www.ncbi.nlm.nih.gov/geo/) and ArrayExpress (https://www.ebi.ac.uk/arrayexpress/). These microarray data sets satisfied the following criteria: organism with “Homo sapiens” and study type with “Expression profiling by array,” MS patients and healthy controls with whole-blood samples or samples of PBMCs, and >20 samples. Data sets representing different types of studies, the relationship between MS patients and other subjects, and/or the therapeutic responses of patients who had received immunosuppressive agents were excluded after the initial evaluation based on the “title,” “overall design,” and “summary.” Those with microarray data from organisms other than Homo sapiens, micro RNA or circular RNA data, or data derived from studies that lacked healthy controls were also excluded after reviewing the “sample characteristics” and “data set details.” After applying the above criteria, the gene expression microarray data sets GSE17048, GSE21942, GSE41890, GSE59085, E-MTAB-69, E-MTAB-4890, and E-MTAB-5151 were downloaded for differential expression analysis and are presented in Table 1 (10–14). Moreover, an independent data set (GSE15245) was downloaded with complete follow-up information for further analysis (9). An integrated data set was obtained with batch normalization. It was randomly classified into a training group for model development (2/3, *n* = 63) and a test group for external validation (1/3, *n* = 31).

**Table 1:** Summary of seven microarray data sets that met the inclusive criteria in this study.

**Accession**	**Released date**	**Platform**	**Source**	**Samples**	
				**Healthy control**	**Multiple sclerosis**
E-MTAB-69	Dec 2, 2009	A-AFFY-44	PBMC	18	26
E-MTAB-4890	Sep 30, 2017	A-MEXP-931	PBMC	40	142 (49 RRMS, 21 SPMS, 23 PPMS, 49 CIS)
E-MTAB-5151	Sep 30, 2017	A-MEXP-931	PBMC	27	49 (21 RRMS, 13 SPMS, 23 PPMS)
GSE17048	Jul 10, 2009	GPL6947	Blood	45	99 (36 RRMS, 20 SPMS, 43 PPMS)
GSE21942	May 20, 2010	GPL570	PBMC	15	14
GSE41890	Oct 28, 2012	GPL6244	PBMC	24	44
GSE59085	Jul 3, 2014	GPL570	PBMC	7	15 (15 CIS)

We downloaded the annotation files to convert the identification probes to gene symbols. The median ranking value was used to compute gene expression if several probes matched one gene. The robust multi-array average was used to log_2_-transform the gene expression values with the *affy* and *affyPLM* packages. The k-nearest neighbors algorithm was used to find and replenish missing values via the *impute* package if necessary.

### Batch Normalization and DEG Identification

Batch normalization is a widely used technique for working with large batches of uncorrelated statistical data in deep neural networks (17). It was used to correct backgrounds, normalize gene expression values, and merge the data sets to reduce error and increase the sample size. The result was a single gene expression matrix. DEGs in MS patients and healthy controls were identified with the *limma* package. The cutoffs were set at log_2_ |fold change| > 0.5 and false discovery rate < 0.05 in the differential expression analysis.

### Gene Functional Analyses

The Gene Ontology (GO) knowledgebase is an open bioinformatics resource that provides functional annotation of gene products in terms of biological process, cellular components, and molecular function (18). The Kyoto Encyclopedia of Genes and Genomes (KEGG) is a public resource for manually integrating data on molecular interaction, reaction, and relation networks of genes and proteins (19). GO and KEGG analyses of these DEGs were performed with the *org.Hs.eg.db, clusterProfiler*, and *GOplot* packages. GO terms and KEGG pathways were considered significant enrichments with a false discovery rate < 0.05. The Search Tool for the Retrieval of Interacting Genes (STRING) database can be used to predict physical and functional associations among different proteins for a wide range of applications (20). Protein–protein interaction (PPI) networks provide reasonable and reliable data on molecular function to help researchers better understand biological mechanisms. We uploaded the DEGs to the STRING database to construct a PPI network and screen hub nodes with high confidence (>0.7).

### Construction of a Co-Expression Network, Identification of Significant Modules, and Selection of Candidate Genes

Weighted correlation network analysis (WGCNA) was used to build a co-expression network of DEGs (21). Pairwise genes were screened with Pearson's correlation matrices. The power function a_xy_ = |c_xy_|^β^ (c = Pearson's correlation, a = adjacency) was used for the weighted adjacency matrix. We obtained the topological overlap matrix by choosing the appropriate parameter β (22). Gene modules were formed through average linkage hierarchical clustering in terms of a topological overlap matrix-based dissimilarity measure with a minimum size of 5. Module eigengenes and module significance were used to identify modules of interest related to clinical traits (23). Then the module connectivity and significance of the clinical traits were used to identify the hub genes. In this study, candidate genes were considered common hub genes between the WGCNA and PPI networks.

### Development and Validation of a Relapse-Free Survival (RFS) Signature

Prognostic genes associated with RFS were selected from the candidate genes with univariate Cox regression analyses in the training group. The optimal predictors from these genes were selected via least absolute shrinkage and selection operator (LASSO)–penalized Cox regression analysis with the *glmnet* and *survival* packages (24). These genes related to RFS were chosen to calculate the risk score for developing the predictive gene signature by nonzero coefficients in the LASSO regression model: risk score = ∑Coef_i_ × *X*_i_ (Coef = coefficient, *X* = serum gene expression). Then the median risk score was used as a cutoff for classifying MS patients into high- and low-risk groups according to their individual scores (25). The Kaplan–Meier method was performed to assess the overall RFS between groups. The area under the receiver-operating characteristic (ROC) curve was used to estimate the 1-, 2-, and 3-year performance of this gene signature.

For external validation, the same gene signature was used to calculate individual risk scores in the test group. Similarly, MS patients were classified into high- and low-risk groups, and the performance of this gene signature for predicting RFS was validated with Kaplan–Meier analysis and ROC curves.

### Identification of Independent Prognostic Parameters and Development of a Nomogram

Clinical features and the gene signature were used to identify independent prognostic parameters through univariate and multivariate Cox regression analyses, including age, gender, disease type, and risk score. A nomogram incorporating these independent prognostic parameters and relevant clinical features was constructed with Cox regression modeling to predict the probability of 1-, 2-, and 3-year RFS in MS patients. We evaluated the discrimination of this predictive model with Harrell's concordance index using a bootstrap method with 1,000 resamples (26). In addition, the performance of this nomogram was validated in the test group.

### Gene Set Enrichment Analysis (GSEA)

GSEA is useful for explaining molecular mechanisms and analyzing biological data (27). The predictive gene signature and related genes in both the WGCNA and PPI networks in the training set were chosen for GSEA to better understand the pathogenesis of MS. This analysis was performed on GSEA software (version 4.0.3) based on the REACTOME pathway database (28). The *c2.cp.reactome.v7.0.symbols.gmt* collection was searched to screen the enrichment pathways associated with poor RFS in both the high- and low-risk groups. Significant cutoffs were set at a false discovery rate < 0.05.

### Statistics

R (version 3.6.1) was used for statistical analyses. Statistical significance was set at *p* < 0.05.

## Results

### Identification of DEGs

**Figure 2:**
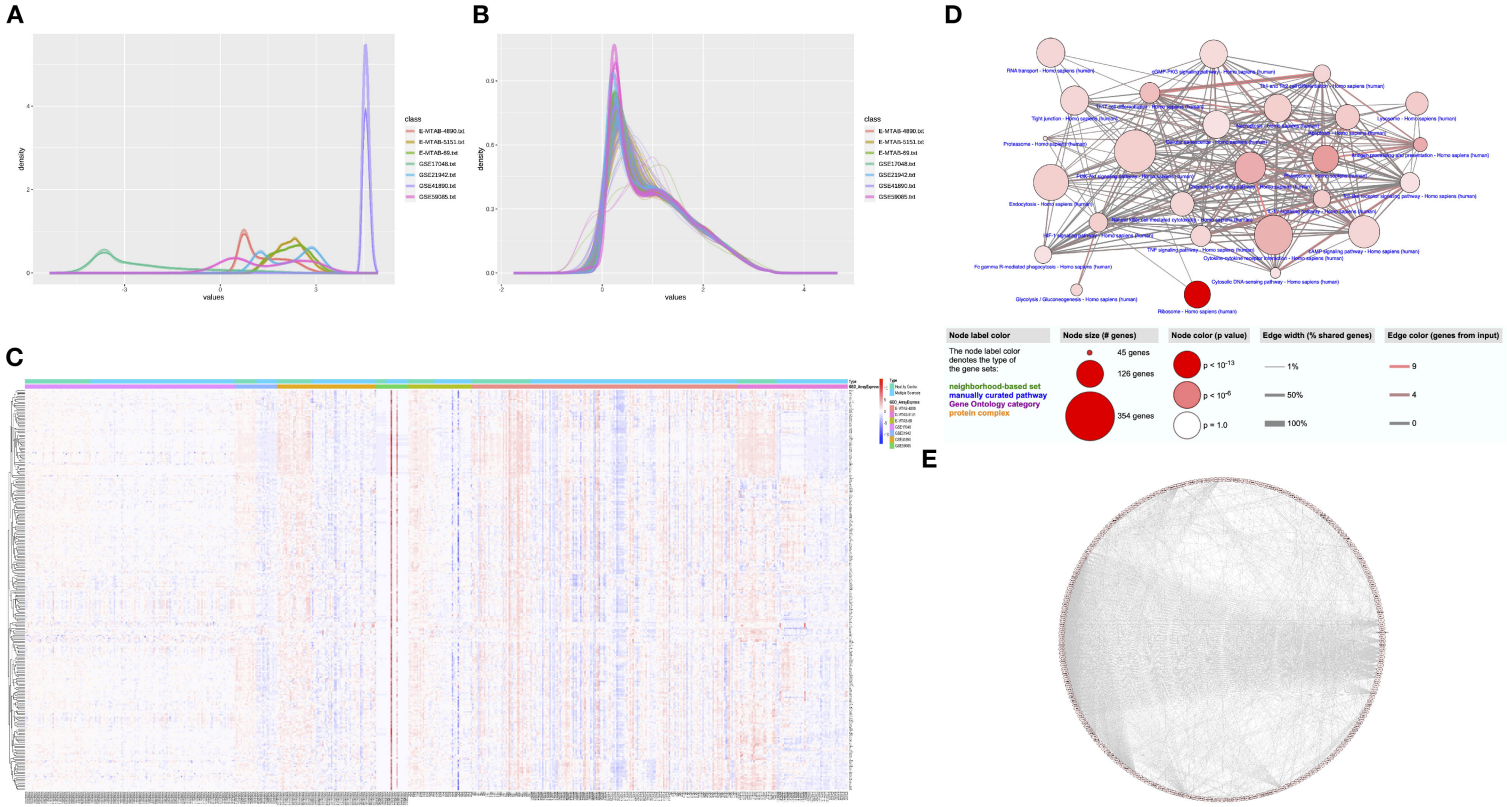
Identification of DEGs and functional analyses. **(A)** The density plot of the seven data sets before normalization. **(B)** The density plot of the seven data sets after batch normalization. **(C)** The heat map of 278 DEGs identified by integrated analysis of the seven data sets. **(D)** Enriched GO terms and KEGG pathways of the DEGs. **(E)** The PPI network of the DEGs. DEG, differentially expressed gene; GO, Gene Ontology; KEGG, Kyoto Encyclopedia of Genes and Genomes; PPI, protein–protein interaction.

The study process is shown in Figure 1B. Details of data sets are available in Gene Expression Omnibus and ArrayExpress. A single gene data set consisting of blood samples from 389 MS patients and 176 healthy controls was obtained through batch normalization for background correction (Figures 2A,B, Supplementary Table 1). A total of 278 DEGs were screened between groups via robust rank aggregation (Supplementary Table 2). The heat map of these DEGs among different data sets is shown in Figure 2C.

### Functional Analyses of DEGs

The results of GO enrichment and KEGG pathway analyses of biological functions are presented in Figure 2D (see also Supplementary Tables 3, 4). Significant biological processes were associated with immune modulation, cell response, metabolism, and cellular hemostasis. The cellular compartments of the DEGs were related to mitochondria, lysosomes, vesicle synthesis, and transportation. The enrichment analysis of molecular functions was relevant to protein modification and cytokine bindings. The KEGG pathway analysis revealed that these DEGs were mainly involved in inflammatory reactions and cellular response, including the antigen processing and presentation pathway, chemokine signaling pathway, phagosome pathway, ribosome pathway, oxidative phosphorylation pathway, and so on. These findings are consistent with previous views that dysfunctions in autoimmune response, cellular metabolism, and autophagy play an indispensable role in MS. The PPI network was built from the STRING database (Figure 2E). A total of 230 genes connected with ≥2 nodes were taken as hub genes for further analysis (Supplementary Table 5).

### Construction of the WGCNA Network, Identification of Modules of Interest, and Screening of Candidate Genes

**Figure 3:**
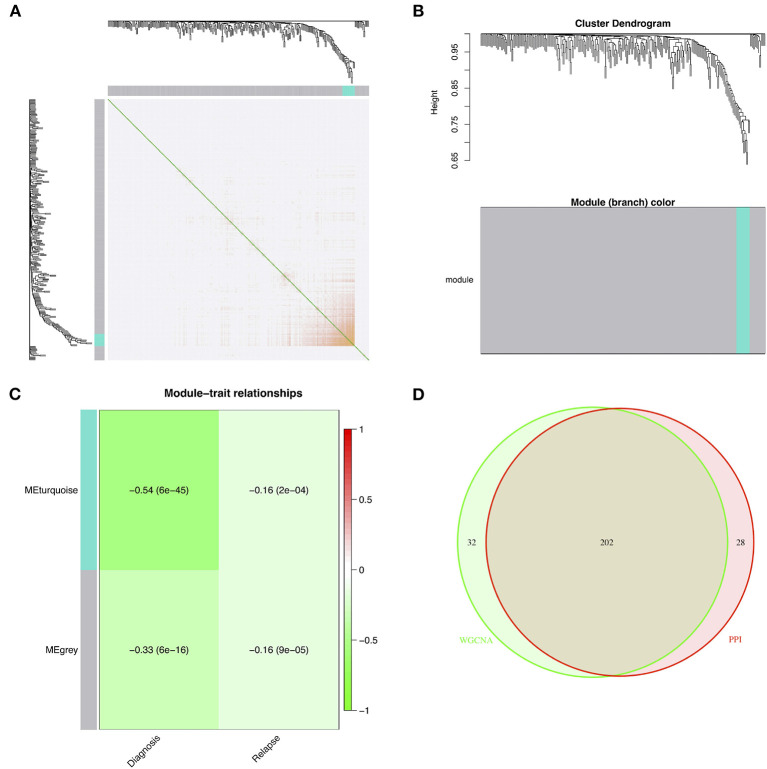
Identification of modules of interest associated with relapse and selected candidate genes. **(A)** The topological overlap matrix after choosing parameter β = 14. **(B)** The dendrogram of all DEGs clustered based on a dissimilarity measure. **(C)** The heat map of the correlations between module eigengenes and clinical features. **(D)** The candidate genes between the PPI and WGCNA networks. DEG, differentially expressed gene; PPI, protein–protein interaction; WGCNA, weighted correlation network analysis.

The DEGs were entered into the WGCNA network through average linkage clustering, and two modules were selected based on β = 14 (Figures 3A,B). Both module eigengenes and module significance were used to determine correlations between modules and clinical features. Both gray and turquoise modules were chosen as modules of interest because of their significant correlations with relapse (Figure 3C). A total of 234 genes from the modules were identified from the WGCNA network as hub genes (Supplementary Table 6). Furthermore, 202 common hub genes were identified as candidate genes between WGCNA and PPI networks (Figure 3D).

### Identification of Key Genes Related to RFS and Construction of a Predictive Gene Signature

**Figure 4:**
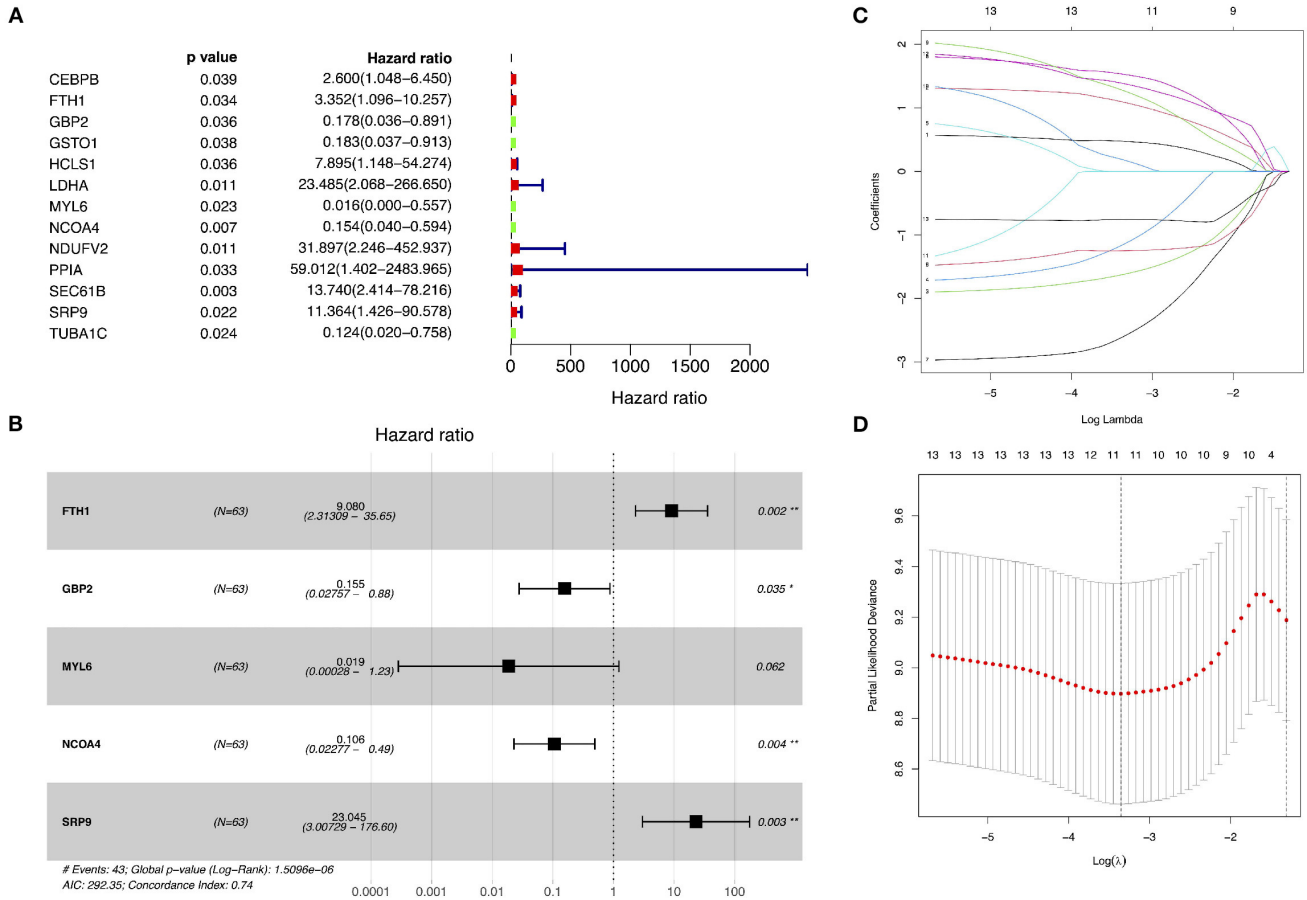
Identification of prognostic genes related to relapse-free survival and development of a predictive gene signature. **(A)** Univariate Cox proportional hazard regression analyses in the training group. **(B–D)** Least absolute shrinkage and selection operator–penalized Cox regression analyses in the training group.

**Figure 5:**
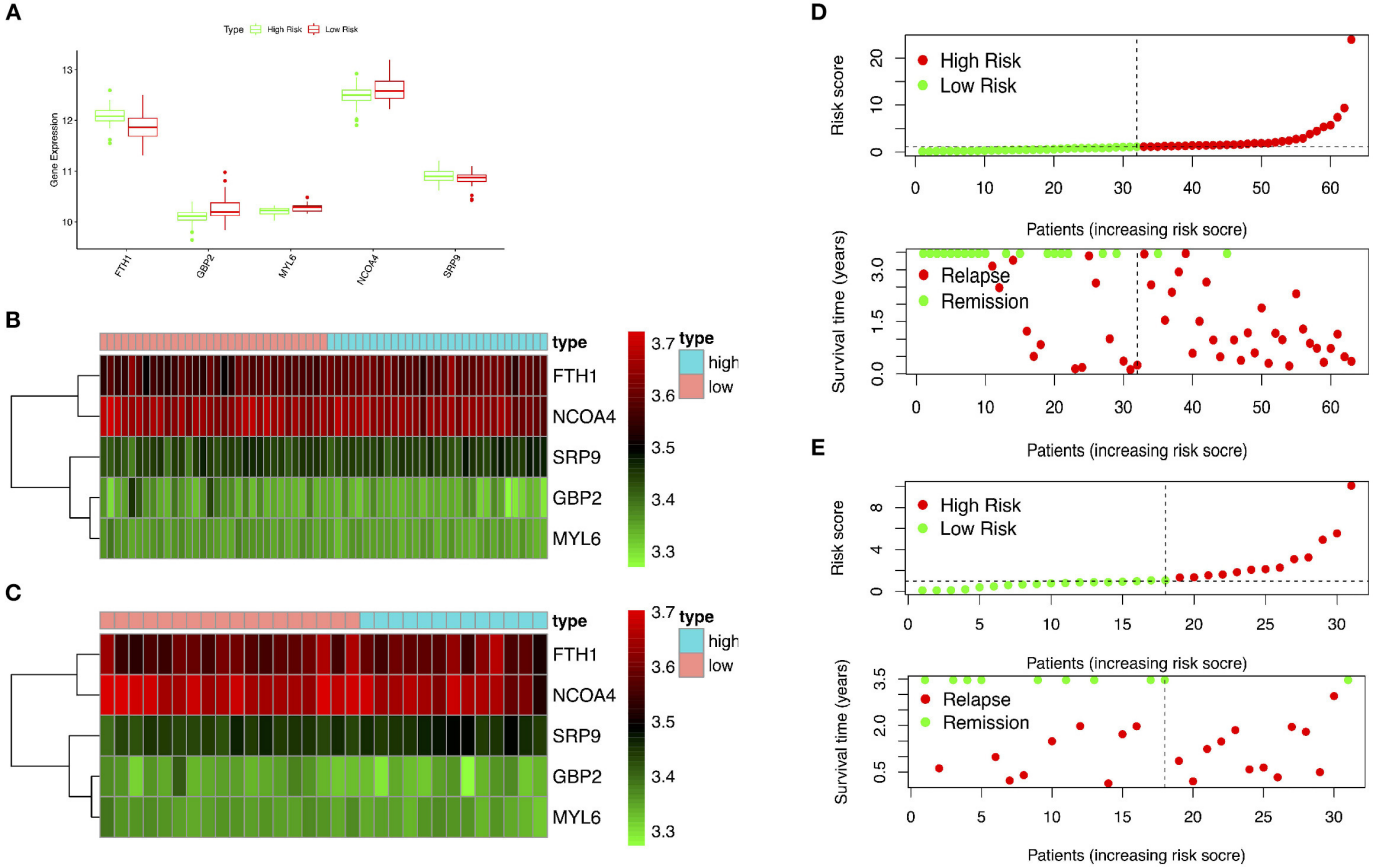
Performance of the gene signature in the training and test groups. **(A,B)** Expression of key genes in high- and low-risk patients in the training group. **(C)** Expression of key genes in high- and low-risk patients in the test group. **(D)** Distribution of risk scores and associated RFS in the training group. **(E)** Distribution of risk scores and associated RFS in the test group. RFS, relapse-free survival.

**Figure 6:**
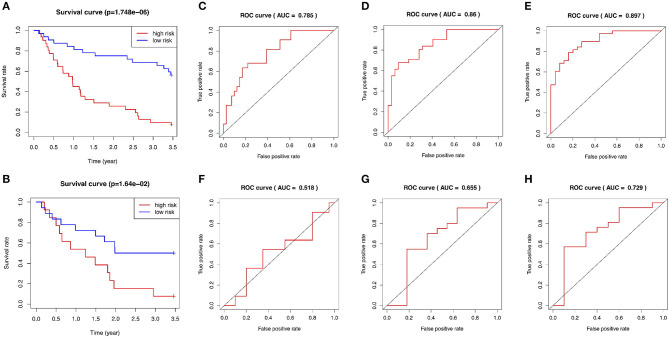
Evaluation of the performance of the five-gene signature in both the training and test groups. **(A)** Kaplan–Meier plots of the five-gene signature in the training set. **(B)** Kaplan–Meier plots of the five-gene signature in the test set. **(C–E)** ROC curves for predictions of 1-, 2-, and 3-year overall RFS in the training set. **(F–H)** ROC curves for predictions of 1-, 2-, and 3-year overall RFS in the test set. ROC, receiver-operating characteristic; AUC, area under the curve; RFS, relapse-free survival.

The GSE15245 data set, with an average follow-up of more than 3 years, was randomized into a training group for developing the gene signature (2/3, *n* = 63) and a test group for external validation (1/3, *n* = 31). The clinical features of the patients are shown in Table 2 (Supplementary Table 7). A total of 202 candidate genes were used to screen the prognostic genes significantly related to overall RFS in the training group via univariate Cox regression analyses, and 13 prognostic genes were identified for further analysis (Figure 4A). Subsequently, a predictive gene signature comprising five genes—ferritin heavy chain (FTH1), guanylate-binding protein 2 (GBP2), myosin light polypeptide 6 (MYL6), nuclear receptor coactivator 4 (NCOA4), and signal recognition particle 9 kDa protein (SRP9)—was developed with LASSO-penalized Cox analysis (Figures 4B–D). Individual risk scores were calculated based on the gene expression and risk coefficient of each gene (Supplementary Table 8). These scores were used to predict the probability of relapse in MS patients and to separate patients into high- and low-risk groups based on the median risk score [1.12, interquartile range (IQR) = 1.11]. Differences in gene expression between groups are shown in Figures 5A–C. Kaplan–Meier plots suggested that the low-risk group benefited significantly in terms of overall RFS compared to the high-risk group (Figure 6A). The number of relapsing patients increased, and the remission period decreased, as the risk score increased (Figure 5D). ROC curves were used to determine the performance of the five-gene signature; the 1-, 2-, and 3-year areas under the curve were 0.785, 0.860, and 0.897, respectively (Figures 6C–E). In short, this gene signature was good at predicting overall RFS in MS patients.

**Table 2:** The clinical features of MS patients in the training group and test group.

**Feature**	**Training (*n* = 63)**	**Test (*n* = 31)**	***p***
**Age (years)**			
18–50	53 (84.1%)	28 (90.3%)	0.413
>50	10 (15.9%)	3 (9.7%)	
Female	41 (65.1%)	19 (61.3%)	0.719
**Type**			
CIS	19 (30.2%)	13 (41.9%)	0.257
MS	44 (69.8%)	18 (58.1%)	
DMT	16 (25.4%)	13 (41.9%)	0.103
Follow-up (years)	2.0 ± 1.3	1.9 ± 1.3	0.182
Relapse	43 (68.3%)	21 (67.7%)	0.960

*MS, multiple sclerosis; CIS, clinically isolated syndrome; DMT, disease-modifying therapy*.

### External Validation of the Gene Signature

The test group was used to validate the prediction performance of the gene signature. We computed a risk score for each patient using the gene signature, and then classified patients into high- and low-risk groups just as we had with the training group (Supplementary Table 9). Kaplan–Meier curves revealed a significant difference in overall RFS between the high- and low-risk groups: The high-risk group showed markedly poorer outcomes (Figure 6B). Predictive ability was assessed with ROC curves (Figures 6F–H). The 1-, 2-, and 3-year areas under the curve for the five-gene signature in predicting RFS were 0.518, 0.655, and 0.729, respectively. Patients in low-risk group had a significantly lower risk of relapse and longer remission than those in high-risk group (Figure 5E). In general, the external validation demonstrated that this predictive gene signature was good at predicting RFS in MS.

### Evaluation of Risk Factors in MS

**Figure 7:**
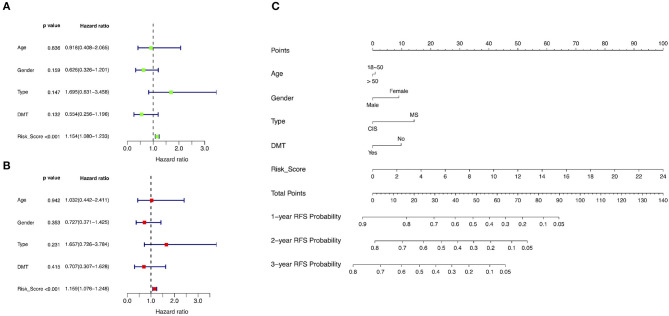
Identification of risk factors associated with RFS and development of a predictive nomogram. **(A)** Univariate Cox regression analyses in the training group. **(B)** Multivariate Cox regression analyses in the training group. **(C)** A novel nomogram predicting the probability of 1-, 2-, and 3-year RFS. RFS, relapse-free survival; DMT, disease-modifying therapy; MS, multiple sclerosis; CIS, clinically isolated syndrome.

A total of 63 patients in the training group with complete clinical data were included for univariate and multivariate Cox regression analyses. The risk score was significantly correlated with overall RFS in the univariate Cox regression analyses (Figure 7A). The multivariate Cox regression analyses also found that the risk score was an independent risk factor associated with overall RFS (Figure 7B).

### Development and Validation of the Predictive Nomogram

The training group was used to develop a novel model consisting of age, gender, disease type, and risk score to predict the probability of 1-, 2-, and 3-year overall RFS based on Cox regression modeling (Figure 7C). The concordance index of this predictive nomogram was 0.67 in the training group. It was 0.59 in the test group in the external validation.

### GSEA

**Figure 8:**
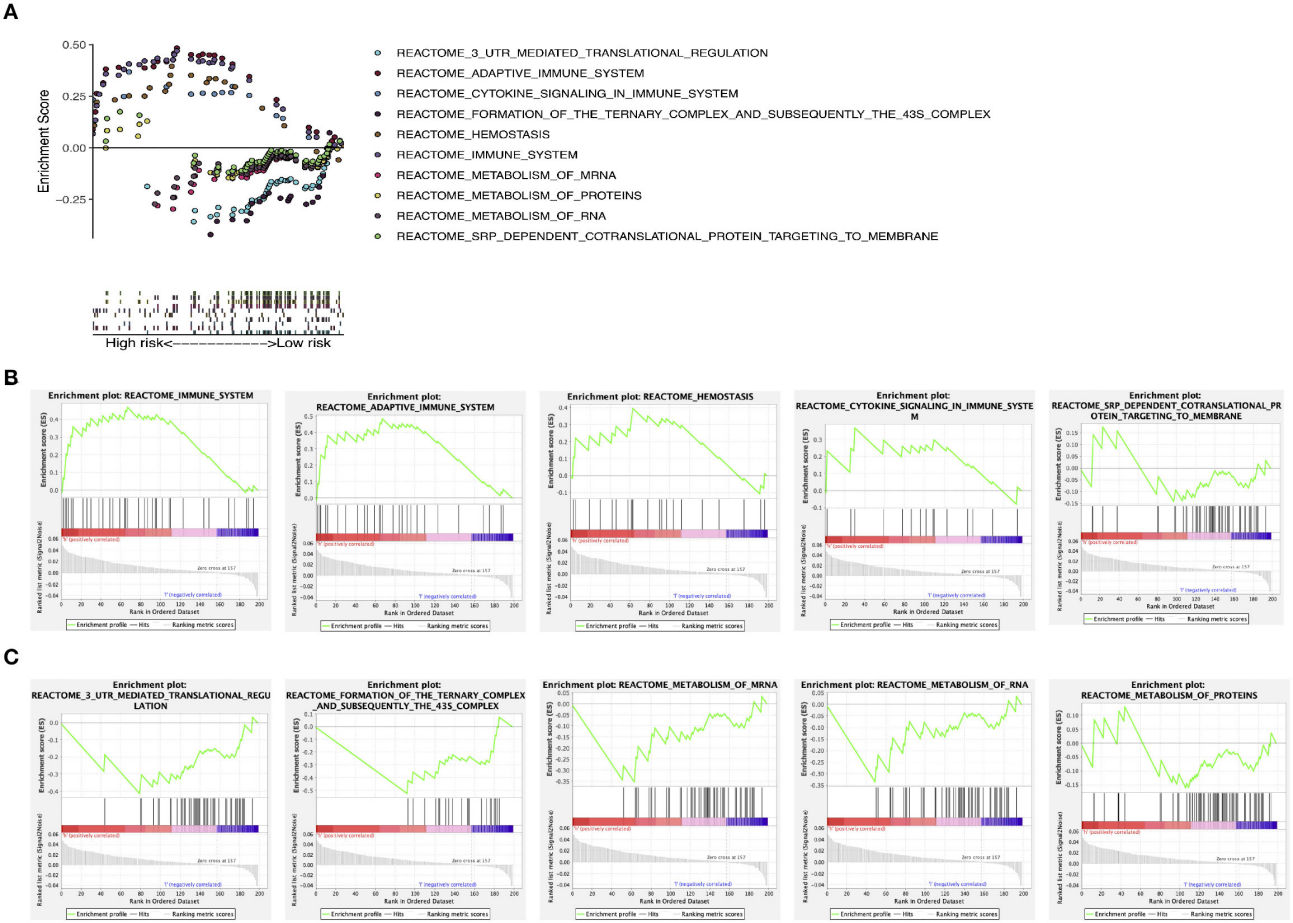
GSEA. **(A)** GSEA of molecular pathways. **(B)** GSEA in the high-risk group. **(C)** GSEA in the low-risk group. GSEA, gene set enrichment analysis.

GSEA found that these prognostic genes and related genes were significantly enriched in several molecular pathways (Figure 8A). In the high-risk group, they were significantly related to the immune system, the adaptive immune system, cytokine signaling in the immune system, hemostasis, and SRP-dependent co-translational protein targeting to membrane (Figure 8B). In the low-risk group, they were significantly associated with 3-UTR-mediated translational regulation, formation of the ternary complex and subsequently the 43S complex, metabolism of mRNA, metabolism of RNA, and metabolism of proteins (Figure 8C).

## Discussion

We investigated whether changes in gene expression in peripheral blood or PBMCs have value for predicting relapses of MS. To do this, we used an integrated analysis and advanced statistics to detect 278 DEGs from seven microarrays with batch normalization. These genes were enriched in immune modulation, cell response, metabolism, and cellular hemostasis according to GO and KEGG pathway analyses. A total of 202 candidate genes were screened through a combination of WGCNA and PPI networks. Then an independent data set (GSE15245) was randomized into a training group (2/3, *n* = 63) and a test group (1/3, *n* = 31). Survival analyses found 13 genes associated with overall RFS in the training group. FTH1, GBP2, MYL6, NCOA4, and SRP9 were downregulated and selected as common prognostic genes between the groups. A five-gene signature was constructed with LASSO-Cox regression to predict RFS in MS patients. Cox regression analyses were also used to develop a novel nomogram that included the gene signature and clinical features; this nomogram was able to precisely predict 1-, 2-, and 3-year overall RFS. In addition, good performance of this gene signature and nomogram was evaluated and validated in both the training and test groups. GSEA revealed that cytokine signaling pathways and immune responses were associated with high risk, whereas the metabolism of RNA and proteins was related to low risk. Indeed, our results showed that this gene signature could be a useful tool in predicting overall RFS in clinical practice.

Similar to a previous study, we identified FTH1 as significantly associated with a relapse of MS (29). FTH1, a key protein in ferroxidase activity, has antioxidant effects and delivers iron to cells. Wang et al. found that FTH1 could be a potential target of the active metabolite dimethyl fumarate, which is remarkably efficacious at reducing relapse rates and protecting neurons from oxidative damage via regulation of the ERK1/2 MAPK signal pathway (29). Furthermore, Dunham et al. (30) developed an accurate marmoset experimental autoimmune encephalomyelitis model showing oxidative injuries within the hallmark of active demyelinating lesions. Thus, dysfunction in the metabolism of oxidative stress might be involved in the pathogenesis of MS.

Unlike previous studies, our study revealed that GBP2, NCOA4, SRP9, and MYL6 are key prognostic genes related to overall RFS in MS. GBP2 in the GTPase family is associated with cellular apoptosis in interferon stimuli. Miao et al. (31) found that highly expressed GBP2 is associated with increased neuronal apoptosis and delayed neurological recovery after traumatic brain injury. Current evidence suggests that it has anti-inflammatory effects by activating the AIM2 inflammasome (32). NCOA4 is a selective cargo receptor that cooperates with ATG8 in autophagy. Mancias et al. (33) found that it plays a key role in ferritinophagy. Also, a lack of NCOA4 leads to a reduction in bioavailable intracellular iron (33). In addition, NCOA4 is a therapeutic target in human cancer. Bellelli et al. (34) found that downregulated NCOA4 improves the sensitivity of DNA-damaging agents for cancer cells. SRP9 is a crucial molecule in signal recognition particle assembly for targeting secretory proteins to the rough endoplasmic reticulum membrane. Along with SRP14 and Alu it forms the ribosome-stalling conformation. It is significantly enriched in ribosome function and neoplasm (35, 36). MYL6 is an ATPase cellular motor protein that is highly expressed in obesity, asthma, and cervical cancer, but the potential mechanisms are not fully known (37, 38). These findings suggest that dysfunctions in inflammatory response, neuron apoptosis, autophagy, and the endoplasmic reticulum might participate in the MS pathology, speculation that should be validated in the future.

Relapse is often accompanied by serious disability and death. Long-term glucocorticoid therapies, immunosuppressive agents, and disease-modifying therapies cannot effectively prevent future relapses in MS patients. Here we developed and validated a novel gene signature to predict the individual risk of relapse in MS patients. We hoped that this signature might precisely classify patients into high- and low-risk groups and evaluate 1-, 2-, and 3-year overall RFS. Our results suggest that the microarray can indeed be used to measure a patient's gene expression changes in peripheral blood or PBMC samples. Next, we developed the following equation using the five-gene system to calculate individual risk scores: risk score = FTH1 × 2.20612057 + GBP2 × (−1.86214371) + MYL6 × (−3.98367166) + NCOA4 × (−2.24490633) + SRP9 × 3.13745827. Using this equation, we determined that the normal risk value would be a median value of 1.12 with an IQR of 1.11. Next, we confirmed this by testing it against real patients. One patient who presented with an FTH1 of 11.0352449, GBP2 of 9.16016007, MYL6 of 9.23298478, NCOA4 of 11.5240002, and SRP9 of 9.92136955 was calculated to have a risk score of 1.47, representing a high risk of relapses; indeed, this patient relapsed in 178 days (Supplementary Table 8). Another patient who presented with an FTH1 of 10.7886453, GBP2 of 9.50607014, MYL6 of 9.3180747, NCOA4 of 11.4456997, and SRP9 of 9.90380955 was calculated to have a risk score of 0.36, representing low risk; this patient has not yet relapsed and 3 years have passed (Supplementary Table 8). For patients at high risk, personalized therapy, lifestyle intervention, and more frequent outpatient follow-ups are needed. In addition, the genes discussed above can act as key molecules in the mechanism of MS and provide insights into relevant biological and signaling pathways, such as antioxidation, immune reaction, apoptosis, autophagy, and so on. They can also be therapeutic targets for drug discovery.

Our study has several strengths. First, we expanded sample sizes and corrected background deviations via batch normalization to identify DEGs from seven microarray data sets. Second, we developed the predictive gene signature from blood samples, an approach that is noninvasive and has clinical applications. In the end, external validation revealed that the results were reliable and had clinical utility. However, this study also has several limitations. The gene expression data and clinical information were all taken from public databases. The development and verification of the gene signature and nomogram were based on one independent data set (GSE15245). Thus, these results must be verified with an external validation cohort with complete clinical and gene expression data. Furthermore, because most of the patients in the sample were Westerners, the results may not generalize to Asians. A study that includes Asians is needed to prove the effectiveness of our gene signature. Finally, transcription and transportation from genes to proteins are involved in regulating non-coding RNA and epigenetic modifying. Differences in the expression of proteins and their molecular mechanisms must be elucidated in further experimental studies.

In conclusion, this study revealed that a five-gene signature is a reliable and noninvasive tool for predicting overall RFS in MS patients. This finding could assist doctors in selecting personalized treatment for patients with MS.

## Data Availability Statement

The datasets presented in this study can be found in online repositories. The names of the repository/repositories and accession number(s) can be found in the article/Supplementary Material.

## Author Contributions

FY designed the study, performed data analysis, and wrote the manuscript. JLia collected the data, performed data analysis, and wrote the manuscript. JLi interpreted the data and created the figures. HL performed the literature search. WS designed the study and revised the manuscript. All authors contributed to the article and approved the submitted version.

## Conflict of Interest

The authors declare that the research was conducted in the absence of any commercial or financial relationships that could be construed as a potential conflict of interest.
